# Optical Properties of Titania Coatings Prepared by Inkjet Direct Patterning of a Reverse Micelles Sol-Gel Composition

**DOI:** 10.3390/molecules200814552

**Published:** 2015-08-12

**Authors:** Veronika Schmiedova, Petr Dzik, Michal Vesely, Oldrich Zmeskal, Magdalena Morozova, Petr Kluson

**Affiliations:** 1 Faculty of Chemistry, Brno University of Technology, Purkynova 118, Brno 61200, Czech Republic; E-Mails: dzik@fch.vutbr.cz (P.D.); vesely-m@fch.vutbr.cz (M.V.); zmeskal@fch.vutbr.cz (O.Z.); 2 Institute of Chemical Process Fundamentals of the ASCR, Rozvojova 135, Prague 16502, Czech Republic; E-Mails: morozova@icpf.cas.cz (M.M.); Kluson@icpf.cas.cz (P.K.)

**Keywords:** material printing, inkjet, ellipsometry, titanum dioxide, optical properties

## Abstract

Thin layers of titanium dioxide were fabricated by direct inkjet patterning of a reverse micelles sol-gel composition onto soda-lime glass plates. Several series of variable thickness samples were produced by repeated overprinting and these were further calcined at different temperatures. The resulting layers were inspected by optical and scanning electronic microscopy and their optical properties were investigated by spectroscopic ellipsometry in the range of 200–1000 nm. Thus the influence of the calcination temperature on material as well as optical properties of the patterned micellar titania was studied. The additive nature of the deposition process was demonstrated by a linear dependence of total thickness on the number of printed layers without being significantly affected by the calcination temperature. The micellar imprints structure of the titania layer resulted into significant deviation of measured optical constants from the values reported for bulk titania. The introduction of a void layer into the ellipsometric model was found necessary for this particular type of titania and enabled correct ellipsometric determination of layer thickness, well matching the thickness values from mechanical profilometry.

## 1. Introduction

Titanium dioxide has been attracting the attention of researchers for the past several decades, it is probably the most extensively studied transition metal oxide [1] because of its unique photo-induced properties, high refractive index, transmittance in the visible spectral range and general chemical stability. It is an excellent semiconductor material, which crystallizes in three different structures: anatase (tetragonal), rutile (tetragonal) and brookite (orthorhombic) [2]. The rutile phase is studied mainly in terms of its optical properties. Recently, the optical and electrical properties of anatase and rutile showed that the major difference between the two phases is the greater optical band gap and a smaller electron effective mass of anatase compared with the rutile phase [3]. Rutile has one of the largest refractive indexes, and also has high dispersion. Anatase phase can develop during crystallization temperatures below 600 °C, while at higher temperatures it is converted to the more stable rutile form. The low-temperature anatase phase is interesting primarily for its efficient electron-hole generation upon irradiation by UV and corresponding photocatalytic effects [4]. Rutile is stable at high temperatures, shows weaker photoactivity and is primarily used for functional coatings in optics, photonics, microelectronics *etc.* TiO_2_ thin films have particular application potential due to its specific properties such as chemical stability, high refractive index, high dielectric constant and transparency in the visible region of the spectrum [2].

Spectroscopic ellipsometry (SE) is a nondestructive optical method that allows the study of optical properties of materials forming thin layers. This technique is based on measuring the state of polarization of the incident and reflected light waves [5]. There is no need to modify the samples as for optical profilometry or interferometry, in the case of which the layer must be reflective and is therefore usually covered by evaporated aluminum. It is possible to determine the complex refractive index by this method and it is equally suited for nondestructive determination of layer thickness.

TiO_2_ thin layers have been successfully prepared by a number of deposition processes including both gas phase methods (PVD and CVD) and liquid phase ones (dip-, spin- or spray-coating, doctor blade spreading, roller coating *etc.*). Generally, the wet coating techniques constitute a simpler alternative to the vacuum processes requiring sophisticated instrumentation and are therefore favored for large-scale manufacturing. Obviously, a suitable liquid precursor formulations need to be developed for these wet-coating techniques. The sol-gel approach proved to be well suited for this purpose and its advantages and benefits were demonstrated repeatedly [6,7,8,9,10,11,12]. The process usually starts with soluble titanium salts and/or titanium alkoxides which are complexed by suitable chelates, pre-crosslinked by partial hydrolysis and the resulting metastable colloidal sols are then coated onto substrate, gelled and converted into dense or porous oxide layer. Thermal calcination is the most common way to achieve this conversion but low temperature alternatives are available as well [13].

However, recently the traditional wet-coating methods have been replaced to a great extent by modified printing techniques [14]. Of these, inkjet material deposition seems to be especially promising [15]. The technique shares basic principles with conventional inkjet printing, *i.e.*, tiny droplets of a low-viscosity liquid are precisely deposited onto a substrate by means of thermal or piezoelectric printhead. In the case of material printing, the ink is a specially formulated low-viscosity liquid used for transporting a functional component onto the substrate surface. Material printing has been successfully used to deliver a wide variety of functional materials and to form thin films, fine 2D patterns and even 3D structures [16].

The authors of this paper have previously reported the deposition of sols based on reverse micelle templates [17] by inkjet printing. The resulting titania patterns showed interesting sensing properties [18] as well as photocatalytic activity [19] and the material inkjet printing approach was found extremely useful due to its precision, repeatability, inherent patterning capability and efficiency of material consumption. In this paper, we present the results of a further study of reverse-micelles-inkjeted titania focused on the optical properties of these layers.

## 2. Results and Discussion

Reverse micelles sol-gel formulation was successfully printed by a Dimatix 2831 printer (Fujifilm Dimatix Inc., Santa Clara, CA, USA) as a simple rectangular pattern 10 mm × 10 mm on glass. The printing process was repeated up to five times to get different thicknesses of the resulting TiO_2_ layers, producing a 5-patch series for each studied calcination temperature. The resulting layers we can observe by optical microscope and by profilometry are smooth, shiny, clear and transparent, with a strong color tint that is the result of light interference. Inkjet printing proved to be an elegant method for sol delivery to a substrate. It provides a complete control of the deposition process parameters together with an excellent efficiency of precursor use. Moreover, the possibility of precise patterning and the ease of up-scaling make this type of deposition very appealing for the production of photonic devices such as sensors, solar electrochemical cells, patterned coatings, *etc.* [20,21,22,23,24,25].

**Figure 1:**
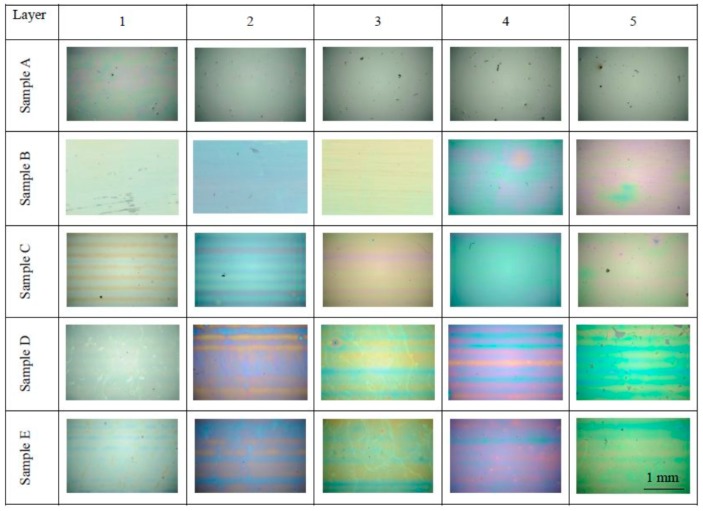
The layer surface as seen by optical microscopy. The matrix shows all the studied samples arranged according to the temperature of processing (rows, sample sets A–E) and according to the number of printed layers (columns, 1–5 layers). At this magnification, the field of view of each photo corresponds to the area 3137 μm × 2083 μm.

Figure 1 shows the visual characteritics of prepared samples as seen in the optical microscope with a 4× magnifying lens. We can observe visually homogeneous surface of B–E series, free of cracks and only rarely spoiled by dust particles.

On some samples, slight banding artifact pattern has developed as the result imperfect band merging during the printing process. However, the A sample series visual appearance is much different, with only traces of interference coloring present at the single layer sample and being apparently much thicker than the corresponding patches from other sample series.

**Figure 2:**
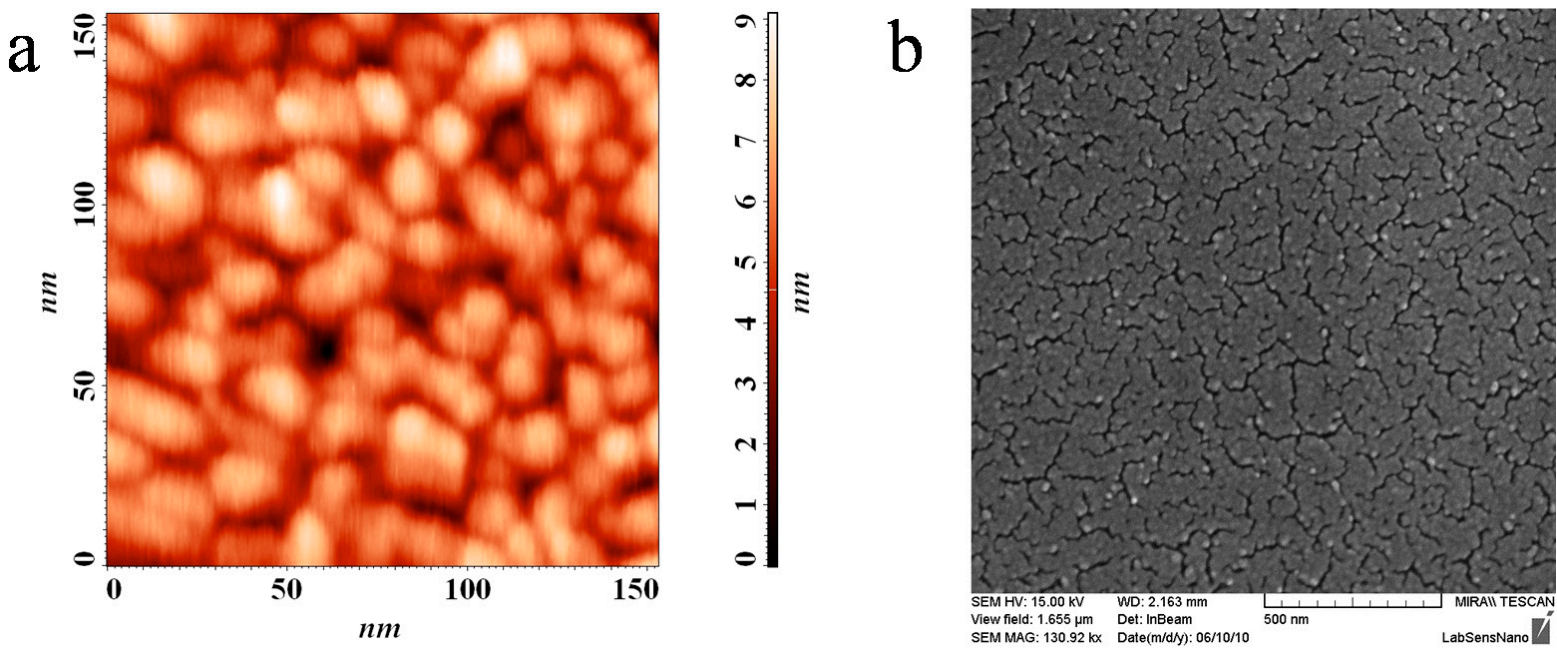
AFM (**a**) and SEM (**b**) scans of the printed layer surface.

We conclude the 200 °C calcination temperature is not sufficient for complete mineralization of the sol and TiO_2_ crystallization therefore this series was omitted from further ellipsometric study (see further discussion of XRD patterns and Table 1 summarizing thickness data for confirmation). Figure 2 shows the SEM and AFM records of the surface layer clearly depicting the micellar template imprints manifested as the globular surface structure known from previous studies of this type of titania layers [26].

**Table 1:** Thickness of prepared samples as determined by ellipsometry.

Samples Layers	A (200 °C)	B (300 °C)	C (400 °C)	D (500 °C)	E (600 °C)
	Thickness (nm)				
1	465.3	89.4	97.9	63.7	56.3
2	918.3	161.9	185.6	150.5	148.1
3	1607.1	251.2	261.2	230.2	229.9
4	2296.5	318.5	331.6	294.8	298.3
5	2670.7	408.7	384.1	353.2	357.4

**Figure 3:**
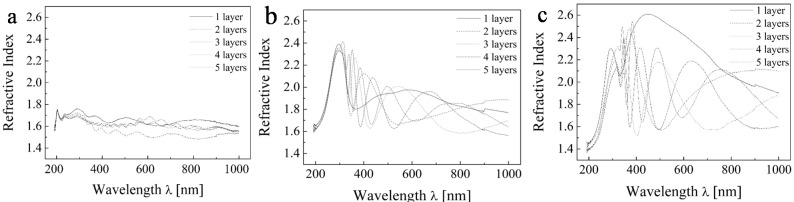
Ellipsometric experimental data of TiO_2_ for the range 200–1000 nm for (**a**) sample A; (**b**) sample C; and (**c**) sample E.

Figure 3 depicts the recorded ellipsometric data expressed in terms of complex refractive index. We can observe the undulating patterns which represent interference and different thicknesses.

**Figure 4:**
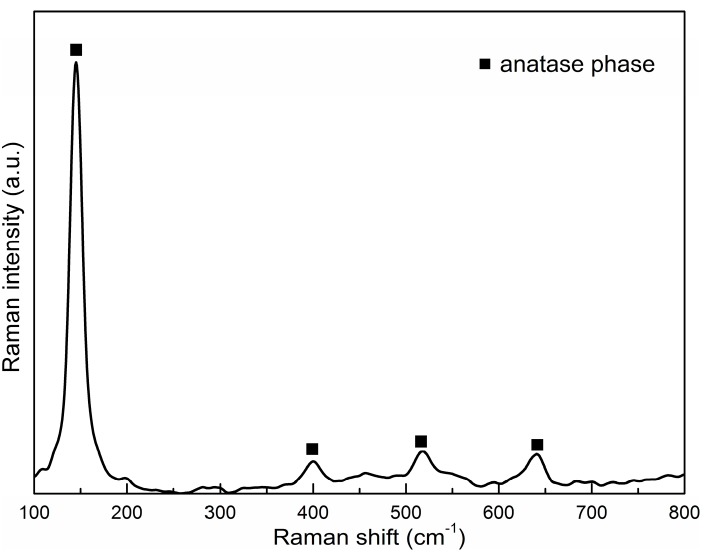
Raman spectra.

We used Raman spectroscopy to detect the crystalline structure (Figure 4, Table 2) of prepared thin films. Our spectrum confirmed the presence of anatase phase. The anatase phase exhibits major peaks at about 635, 514, 396 and 144 cm^−1^.

**Table 2:** Characteristic of the studied TiO_2_ thin layers prepared by sol-gel method.

Sample	Temperature (°C)	Supposed Crystal Phase by Raman Spectroscopy
A	200	incomplete mineralization and/or crystallization
B	300	amorphous
C	400	Anatase
D	500	Anatase
E	600	Anatase

**Figure 5:**
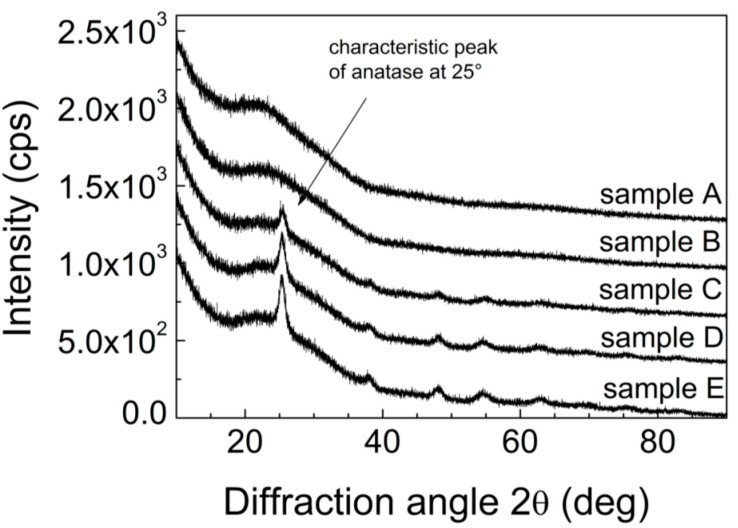
XRD patterns for samples A–E.

Figure 5 shows obtained X-ray diffraction (XRD) spectra for to study the crystallinity of the films that as the annealing temperature increases the intensity and linewidth of the (101) diffraction peak. All the diffraction peaks of TiO_2_ can be indexes as anatas (Anatase XRD JCPDS card no. 78-2486). As expected, with increasing calcination temperature the crystatalinity improves which is manifested by more prominent diffraction peaks of samples calcined at higher temperatures.

### 2.1. Simplest Model of Studied Sample

**Figure 6:**
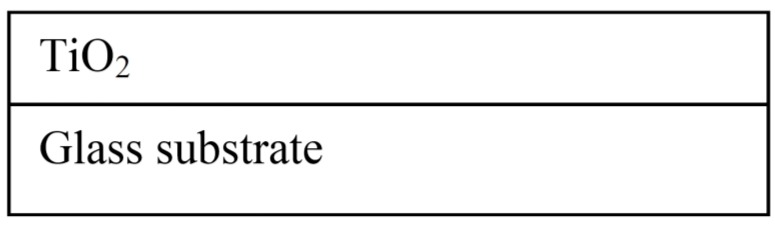
A schematic outline of the used ellipsometric model.

**Figure 7:**
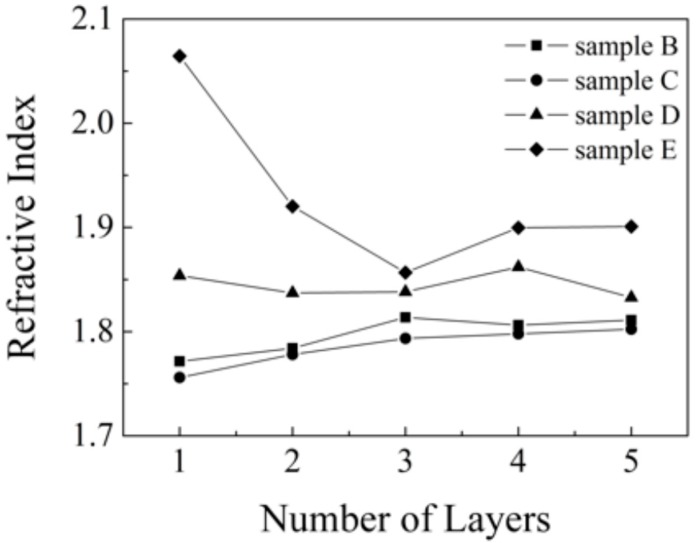
Refractive index on studied samples each of layers at 590 nm wavelength after fitting.

Initially, the ellipsometric spectra were analyzed on the basis of the simplest dispersion model consisting of glass/TiO_2_ layers (Figure 6). As is evident from Figure 7, there are considerable variations between the published results for TiO_2_ optical constants and the results which were obtained using the simple model. For example, the previously reported values of *n* (633 nm) was observed to be in the range of 2.43–2.50 for single anatase crystals obtained by the deposition on the substrate heated to 250 °C [27].

However, the computed thickness values of the layers obtained by fitting this simple model agree with the results obtained using a mechanical profilometer fairly well, with the exception of the thickest sample, where a deviation of 23% was observed. The results of both methods for all samples analyzed (layers 1–5) are given in Table 1 and Table 3.

**Table 3:** Thickness of prepared samples as determined by mechanical profilometer.

Sample Layer	A (200 °C)	B (300 °C)	C (400 °C)	D (500 °C)	E (600 °C)
	Thickness (nm)				
1	288.9	63.3	80.0	96.0	77.9
2	542.2	145.8	153.4	156.5	159.1
3	894.1	196.7	227.5	223.8	216.0
4	1025.6	305.8	307.1	273.1	295.3
5	1752.0	331.0	390.7	389.4	370.6

### 2.2. Complex Model of Studied Samples

**Figure 8:**
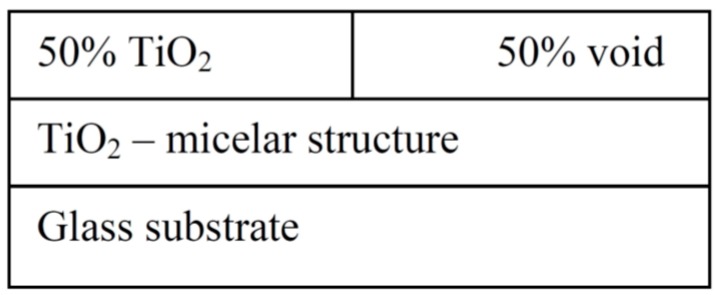
Used ellipsometric model which involves a layer with an additional void.

Since crystalline anatase phase was previously proved to be present in the layers calcined at 400–500 °C [18], but the value the calculated refractive index is not in agreement with optical parameters for TiO_2_, we need to adopts a more complex model for experimental data fitting (Figure 8). This model was suggested on the basis of studying the images from electron and AFM microscopy, where the surface topology clearly manifesting the globular surface structure resulting from the micellar templating became obvious. This new model consist of two layers: the bottom one consists of the compact TiO_2_ inherited from the simple model and a top surface layer consisting of pure TiO_2_ mixed with variable void fraction.

**Figure 9:**
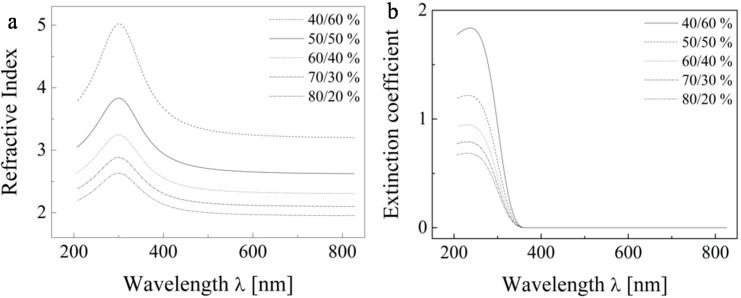
The dispersion dependence of the refractive index (**a**) and extinction coefficient (**b**) of the pure TiO_2_ solid fraction only.

Within this model we investigated two variables: the ratio of thicknesses of the two layers and also the ratio of TiO_2_ and void in the upper layer. First, we studied the fraction effect of the void in the upper layer. For these calculations we finally assumed that 90% of the total thickness is represented by nonporous TiO_2_ and 10% builds up the surface voided layer (the explanation of selecting this particular thickness ratio follows in the paragraph below). We confirmed the expected fact that the void fraction value greatly influences the dispersion dependence of the refractive index on the wavelength. Obviously, with increasing void fraction the global index of refraction of the whole layer system is decreasing. However, if we attempt to substitute a solid layer by a composite one consisting of a solid material and a certain fraction of void, then with increasing void fraction the refractive index of the complementary solid fraction must increase as well in order to keep the global refractive index constant. This situation is modeled at Figure 9. Based on this modeling, we adopted the 50% void fraction as a reasonable starting point.

**Figure 10:**
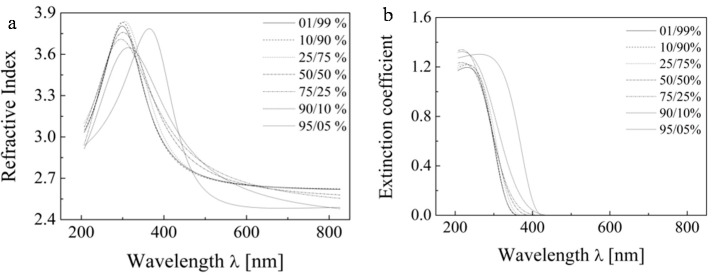
The dispersion dependence of the printed samples for various thickness ratios of the two sublayers of the refractive index (**a**) and extinction coefficient (**b**).

Subsequently, we attempted a further tuning of the ellipsometric model by optimizing the second variable, *i.e.* the thickness ratio of the two layers. The results are presented at Figure 10 for sample B (one layer) and the ratio of TiO_2_/void in the upper layer is set to 50/50% by the previous analysis. The figure shows that the ratios of thicknesses of layers have much smaller role. Nevertheless, the ratio 90/10% was selected because it provided the best match to refractive index values reported for TiO_2_ at 590 nm.

**Figure 11:**
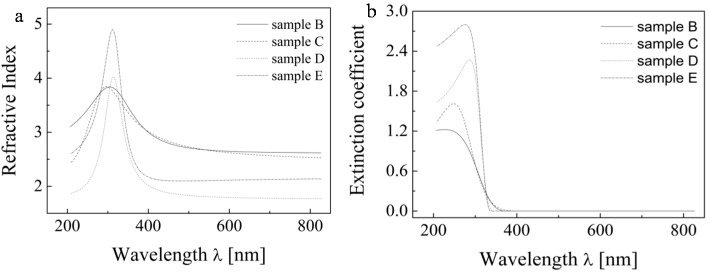
The final computed refractive index (**a**) and extinction coefficient (**b**) values for studied samples on their first layer.

Once the thickness ratio 90/10 was selected, this ellipsometric model was applied to all studied samples. These results are depicted in Figure 11 and Table 4.

**Table 4:** Properties refractive index of prepared materials TiO_2_ by sol-gel method at 590 nm wavelength.

Samples Layers	B (300 °C)	C (400 °C)	D (500 °C)	E (600 °C)
	Refractive Index			
1	2.650	2.628	1.794	2.112
2	2.690	2.705	2.422	2.578
3	2.768	2.717	2.809	2.618
4	2.803	2.764	2.817	2.779
5	2.792	2.811	2.768	2.914

We can see that the refractive index value is in the range of 2.6 to 2.9 for most of our samples, which matches well the reported values. For the sample series calcined at lower temperatures (B, C) we can note slight increase of refractive index values with increasing layer thickness, probably due to better crystallite development in the thicker samples. However, an anomalous decrease of the refractive index value with decreasing layer thickness can be observed in the case of the more heated sample series D and E. We expect that during calcination at elevated temperatures in the proximity of glass softening temperature, a significant material diffusive mixing takes place, this phenomenon has been observed earlier [28] and is known to have a detrimental effect on the photocatalytic performance of titania layers resulting from sodium ions diffusion into titania lattice. It seems that the thinner one- and two-layered samples suffer the most due to the contaminant diffusion path length being comparable to the total layer thickness, while the thicker samples are less sensitive because only a fraction of their thickness is contaminated. Moreover, the negative effect of diffusing contaminants can be eliminated by improved crystallization in the thicker samples.

Obviously, sodium diffusion can be eliminated by using another substrate such as quartz or silicon. Titania coating deposited onto these substrates can be processed at much higher temperatures which would open the pathway to ellipsometric investigation of phase change from anatase to rutile. However, working with different substrates would require the modification of our ellipsometric models and would go far beyond the scope of this paper.

## 3. Experimental Section

Nanostructured micellarly templated TiO_2_ layers were prepared by inkjet printing of our previously optimized [18] sol onto soda-lime glass plates (25 mm × 75 mm) usually used for microscopy. Sol deposition and patterning was performed with a dedicated Fujifilm Dimatix 2831 material inkjet printer. The drop formation characteristics were monitored by means of the built-in stroboscopic camera and the interaction with substrate was inspected by an optical microscope. Optimal printing conditions were defined in the previous studies: Dimatix Model fluid 2 waveform, 20 V driving voltage, nozzle temperature 30 °C, substrate temperature 40 °C, and nozzle span was kept constant 20 µm.

The annealing temperatures after deposition are summarized in Table 2. Printing was repeated several times in a wet-to-wet manner. Step-wedge like samples were fabricated by repeated overprinting onto a single 26 mm × 76 mm glass slide. They consisted of five square patches 10 mm × 10 mm with gradually increasing number of layers. This study started with five parallel samples series denoted as A, B, C, D, and E, all printed in exactly the same way. However, they were calcined at different temperatures (see Table 2) and the duration of the thermal treatment was 4 h. Raman spectroscopy was employed for the determination of crystal phase of the studied samples.

Microphotographs were recorded using Nikon Eclipse E200 optical microscope equipped with a Nikon D5000 digital camera and Nikon Camera Control Pro 2 software (Nikon, Tokyo, Japan). Polarised light was used to enhance the interference-originating color of the printed layers. Captured raw images were conveniently cropped, color balanced and organized by Adobe Lightroom. Scanning electronic microscopy was performed on the MIRA II LMU (Tescan, Warrendale, PA, USA) and AFM analysis was done on the Ntegra Prima by NT-MDT (Santa Clara, CA, USA). Physical thickness of the layers was determined by a Dektak XT mechanical profilometer (Bruker, Tucson, AZ, USA).

A UVISEL 2 spectroscopic ellipsometer [27] by Jobin-Yvon (Palaiseau, France) was used for recording the wavelength dependency of the principal ellipsometric quantities ψ and Δ at the angle of 70°. The setup of the phase modulated ellipsometer is as follows: xenon source (spectral range 1.5–5.5 eV) polarizer/modulator/sample/analyzer/monochromator and detector (photomultiplier). The experimental ellipsometric angles ψ and Δ are a measure of the polarization change of the incident light wave as it is reflected at the different interfaces in the film profile. In order to determine the optical constants, the film thickness and the surface roughness, a model film structure has to be established. In this simple single layer case, the model consists of a glass substrate and a dense TiO_2_ layer. Optional 50% TiO_2_:50% void surface layer, which simulates the surface roughness, may be added to improve the accuracy of modelling and the New Amorphous model was used for this purpose. It's based on Forouhi-Bloomer formulation. The absorption coefficient is given by Equation (1) and refractive index given by Equation (2):

(1) $$k(\omega )=\{\begin{array}{l} \frac{f_{\text{j}}{\left(\omega -\omega_{\text{g}}\right)}^{2}}{{\left(\omega -\omega_{\text{j}}\right)}^{2}+\Gamma_{\text{j}}^{\text{2}}}\\ 0 \end{array}\;\begin{array}{l} \text{;for }\omega >\omega_{\text{g}}\\ \text{;for }\omega \leq \omega_{\text{g}} \end{array}$$

where is $\omega_{\text{j}}=(E_{\sigma *}-E_{\sigma })/\hbar$ difference the basic frequency and the excited state, $\Gamma_{\text{j}}=\gamma /2$ is the broadening term of the peak absorption and coefficient *f*_j_ = *A* is proportional to the strength of the oscillator:

(2) $$n(\omega )=n_{\infty }+\frac{B_{\text{j}}(\omega -\omega_{\text{j}})+C_{\text{j}}}{{\left(\omega -\omega_{\text{j}}\right)}^{2}+\Gamma_{\text{j}}^{\text{2}}}$$

where $B_{\text{j}}=\frac{f_{\text{j}}}{\Gamma_{\text{j}}}\left[\Gamma_{\text{j}}^{\text{2}}-{\left(\omega_{\text{j}}-\omega_{\text{g}}\right)}^{2}\right]$ and $C_{\text{j}}=2\;f_{\text{j}}\;\Gamma_{\text{j}}\;(\omega_{\text{j}}-\omega_{\text{g}})$ are coefficients. Where *n*_∞_ is refractive index for ω → ∞, ω_j_ is the energy at which the extinction coefficient is maximum (peak of absorption), *f*_j_ is related to the strength of the extinction coefficient peak, ω_g_ is the energy band gap and Γ_j_ is the broadening term of the peak of absorption. Recorded spectra in combination with different ellipsometric models were processed in the DeltaPsi software package (HORIBA Scientific Systems, Longjumeau Cedex, France) and a complete optical characterization was attempted.

Since both the printed TiO_2_ layers and the glass substrates are transparent, it is important to suppress the backside reflections [29] from transparent glass substrates. These unwanted backside reflections must be accounted for in the fit model or suppressed by experimental arrangement. A common technique used to eliminate backside reflections is to focus the probing beam onto an area small enough to permit the separation of the front and the back reflection. Alternatively, the reflections can be suppressed by modifying the reverse side of substrate opposite the measurement spot. This can be accomplished by mechanical grinding using a fine sandpaper or, as in our case, painting the reverse side by black marker.

## 4. Conclusions

The paper presents the results of a comprehensive study on the optical properties of TiO_2_ layers consisting of micellarly templated TiO_2_ fabricated by material printing. We studied the influence of the ratio of thicknesses of the both layers and void fraction effects on the optical properties of the layer forming material. We demonstrate that the influence of voids on the properties of the layer is substantial—at 50% void fraction the refractive index of the complementary fraction increased 1.8 times (from 1.7 to 2.4 for a wavelength of 590 nm). On the other hand, the influence of the ratio of thicknesses of the upper and lower layers was not so critical. Nevertheless, the ratio 90%/10% was selected because it provided the best match to refractive index values reported for TiO_2_ at 590 nm. After the incorporation of these two variables into the ellipsometric model, a reasonably good agreement between calculated and tabulated values of the refractive index was obtained.
